# Assessment of the Concomitance of Fibromyalgia in Patients with Axial Spondyloarthritis and Rheumatoid Arthritis

**DOI:** 10.5152/ArchRheumatol.2026.25240

**Published:** 2026-01-16

**Authors:** Mehmet Caglayan, Pelin Oktayoglu, Yusuf Celik, İbrahim Gunduz, Zeynep Işık Sula, Mehmet Ciftci, İsmail Yıldız

**Affiliations:** 1 Department of Physical Medicine and Rehabilitation, Dicle University Faculty of Medicine, Diyarbakır, Türkiye; 2 Department of Biostatistics, Biruni University Faculty of Medicine, İstanbul, Türkiye; 3 Department of Rheumatology, Diyarbakır Selahattin Eyyubi Education and Research Hospital, Diyarbakır, Türkiye; 4 Department of Biostatistics, Dicle University Faculty of Medicine, Diyarbakır, Türkiye

**Keywords:** Axial spondyloarthritis, biologic agents, fibromyalgia, rheumatoid arthritis

## Abstract

**Background/Aims:**

Fibromyalgia (FM) is a chronic pain condition in which patients experience diffuse musculoskeletal discomfort accompanied by marked fatigue, non-restorative sleep patterns, and cognitive complaints. Individuals with FM exhibit elevated inflammatory cytokines. The aim was to investigate the prevalence of FM in patients with rheumatoid arthritis (RA) and spondyloarthritis (SpA) and to evaluate the effects of biologic and targeted synthetic disease-modifying antirheumatic drugs (b/tsDMARDs) on FM.

**Materials and Methods:**

Patients were enrolled in the study, including spondyloarthritis and RA patients. Disease activity in RA patients was assessed using the Disease Activity Score–28 scores. For SpA patients, the Bath Ankylosing Spondylitis Disease Activity Index and the Bath Ankylosing Spondylitis Metrology Index were used to evaluate disease activity and structural damage. Patients were evaluated for FM according to the 1990 American College of Rheumatology classification criteria. The Fibromyalgia Impact Questionnaire was administered for FM severity.

**Results:**

There was no significant difference in the prevalence of FM between the RA and SpA groups (*P* = .942). The prevalence of FM was significantly higher among patients not receiving b/tsDMARDs compared to those who were (*P* = .033). In the SpA subgroup, no significant difference in FM prevalence was observed between patients using and not using b/tsDMARDs (*P* = .314). However, in the RA subgroup, FM prevalence was significantly higher among patients not receiving b/tsDMARDs (*P* = .030).

**Conclusion:**

The findings suggest that the use of b/tsDMARDs appears to be associated with a reduced prevalence of FM among patients with RA. It is believed that this study provides a window for future studies.

**Main Points**

- The prevalence of fibromyalgia (FM) did not differ significantly between the rheumatoid arthritis (RA) and spondyloarthritis (SpA) groups.
- Whereas no significant difference in the frequency of FM is observed between patients with and without biologic and targeted synthetic disease-modifying antirheumatic drug (b/tsDMARDs) use in the spondyloarthritis group, the use of b/tsDMARDs appears to be associated with a reduced prevalence of FM among patients with RA.
- In patients with RA, the status of FM is closely associated with the use of b/tsDMARDs, whereas in patients with SpA, the status of FM is strongly associated with the duration of b/tsDMARD therapy.

## Introduction

Fibromyalgia (FM) is a chronic pain condition in which patients experience diffuse musculoskeletal discomfort accompanied by marked fatigue, non-restorative sleep patterns, and cognitive complaints. Although its biological basis has not yet been clarified in full, accumulating research points to a potential contribution of inflammatory pathways to both the emergence and persistence of FM symptoms.^1^

Spondyloarthritis (SpA) represents a family of inflammatory rheumatic diseases that share certain clinical and genetic features but differ in presentation. This group includes psoriatic arthritis, arthritis associated with inflammatory bowel disorders, reactive arthritis, particular forms of juvenile idiopathic arthritis, and ankylosing spondylitis.^2^ Rheumatoid arthritis (RA), in contrast, is a systemic inflammatory disease primarily targeting synovial joints, where chronic inflammation leads to progressive erosion of cartilage and bone and ultimately to considerable functional limitations. Key mediators such as tumor necrosis factor-α (TNF-α) and various interleukins (IL-1, IL-6, IL-17) orchestrate the inflammatory milieu in RA, promoting activation of macrophages and osteoclast-driven bone destruction.^3^

Although joint inflammation has historically been considered the principal source of pain in RA, numerous investigations show that pain severity does not always correspond to measurable inflammatory activity. This discrepancy suggests that a subset of RA patients may experience concurrent FM, which could account for persistent pain even when inflammatory indices appear well controlled.^4^^,^^5^ A similar pattern is evident among individuals with axial SpA (axSpA): those fulfilling FM criteria typically report higher perceived disease activity, more functional constraints, lower quality of life, greater fatigue, and more frequent psychological symptoms compared to axSpA patients without FM.^6^^-^^9^

Evidence increasingly implicates inflammatory activity within the central nervous system—often referred to as neuroinflammation—in the pathophysiology of FM.^10^ Elevated levels of several pro-inflammatory cytokines have been identified in the cerebrospinal fluid of individuals with FM.^11^^,^^12^ In addition, chronic low-grade systemic inflammation has been proposed as a common biological substrate for FM and other long-standing pain conditions.^13^^,^^14^ Immune cells, which can adapt their functional phenotype depending on environmental cues, may release either pro- or anti-inflammatory mediators. In FM, circulating cytokine levels tend to be increased, and peripheral immune cells display heightened cytokine release upon stimulation.^14^^-^^16^

Several systematic reviews have highlighted that serum concentrations of IL-1 receptor antagonist (IL-1Ra), IL-6, and IL-8 tend to be elevated in FM cohorts,^17^ although studies using peripheral blood mononuclear cells (PBMCs) have yielded inconsistent results.^18^ Variability in study design, population size, PBMC composition, laboratory stimuli, and analytic protocols likely contributes to these divergent findings.

Pro-inflammatory cytokines such as TNF-α, IL-6, and IL-1β are recognized to sensitize nociceptive pathways, evoke pain in human subjects, and induce hyperalgesia in experimental models. Interleukin-6, for example, can render skeletal muscle more responsive to subsequent noxious stimuli, as demonstrated in studies showing enhanced nociceptive reactions following intramuscular prostaglandin E₂ administration in primed tissue.^19^ Furthermore, inflammatory signaling has been linked to stress-related worsening of muscle pain—a frequent complaint among patients with FM.^20^ Collectively, these data indicate that pro-inflammatory cytokines play a substantial role in amplifying and sustaining chronic musculoskeletal pain, including that characteristic of FM.^21^

Against this background, the present study sought to examine whether biologic agents and targeted synthetic disease-modifying antirheumatic drugs (DMARDs), commonly used in the treatment of RA and SpA, influence the severity of FM-related symptoms in patients in whom FM and inflammatory rheumatic disease coexist.

## Materials and Methods

During the period of 2022-2023 and 2024-2025, a total of 218 patients classified as axSpA according to the Assessment of SpondyloArthritis International Society (ASAS) 2010 classification criteria,^22^ and 142 patients classified as RA according to the 2010 American College of Rheumatology/European League Against Rheumatism (ACR/EULAR) classification criteria,^23^ were included in this study. Among the 218 axSpA patients, 21 fulfilled both the ASAS axial and peripheral SpA criteria.^24^ All of these patients were categorized under the SpA group.

Patients were divided into 2 groups: the SpA group and the RA group. Due to a temporary suspension of the study for individual reasons, no patients were enrolled between 2023 and 2024.

Exclusion criteria included a diagnosis of diabetes mellitus, overlap syndromes, polyneuropathy, history of cerebrovascular accident, use of antidepressants, pregabalin, gabapentin, or duloxetine, and a known history of FM. Patients aged 18-80 years who agreed to participate were included in the study. All participants were informed about the study protocol, and both verbal and written informed consent were obtained.

Demographic and clinical data—including age, sex, body mass index, occupation, educational level, disease duration, and current medical treatments—were recorded. The use of conventional synthetic DMARDs (csDMARDs), biologic DMARDs (bDMARDs), targeted synthetic DMARDs (tsDMARDs), and corticosteroids was documented. For both patient groups, the therapies received by patients treated with either biologic or targeted synthetic agents were also noted under the b/tsDMARDs treatment.

In RA patients, tender joint count, swollen joint count, and Disease Activity Score in 28 joints (DAS28) were recorded. In axSpA patients, the presence of peripheral arthritis was noted. Disease activity was evaluated using the Bath Ankylosing Spondylitis Disease Activity Index (BASDAI),^25^ while structural damage was assessed using the Bath Ankylosing Spondylitis Metrology Index (BASMI).^26^

All patients were evaluated for FM syndrome according to the 1990 ACR classification criteria.^27^ The number of tender points was determined, and the coexistence of FM with RA or SpA was recorded. Patients diagnosed with FM completed the Fibromyalgia Impact Questionnaire (FIQ), and their results were documented. The FIQ is an instrument developed to assess the impact of FM on patients’ daily functioning and quality of life.^28^

This study was conducted in line with the ethical principles of the Declaration of Helsinki (1964) and its later amendments. Ethical approval was obtained with the number 187 and date: October 13, 2017, from the medical faculty’s ethics committee for noninterventional studies.

### Statistical Analysis

The distributional characteristics of continuous variables were initially evaluated using the Kolmogorov–Smirnov test. For variables that did not meet the assumption of normality, comparisons between groups were performed using the Mann–Whitney *U*-test, and the results were summarized as medians with interquartile ranges (25th-75th percentiles). For datasets that exhibited a normal distribution, inter-group comparisons were conducted using the Student’s *t*-test, with results reported as means and SDs. Differences in proportions between categorical variables were examined using the chi-square test.

To improve the robustness and internal validity of the analytical framework, multivariate statistical procedures were preferred over solely univariate techniques. In this context, cluster analysis accompanied by dendrogram visualization was employed to reduce the dimensional complexity of the dataset and to identify underlying structures among variables. A two-tailed *P*-value of ≤.05 was considered indicative of statistical significance. All statistical computations were carried out using R software version 4.3.2. (Statistical Computing, Vienna, Austria) and IBM SPSS Statistics for Windows, version 21.0 (IBM SPSS Corp.; Armonk, NY, USA).

## Results

A total of 360 patients were included in the study: 218 with SpA and 142 with RA. Among the SpA patients, 11 were diagnosed with psoriatic arthritis and 3 with enteropathic arthritis. During the study period, 60 axSpA and 70 RA patients met the exclusion criteria and were therefore excluded, leaving 360 patients who were included in the study.

Patients were divided into 2 groups: the SpA group (comprising both axial and peripheral SpA patients) and the RA group. The demographic and clinical characteristics of the study population are summarized in Table 1.

When patients with RA and those with SpA were compared, a statistically significant difference was observed between the 2 groups in terms of age, sex, and height (*P* = .001). However, there was no significant difference between the 2 groups regarding the duration of b/tsDMARDs use (*P* = .304). Similarly, the prevalence of FM did not differ significantly between the RA and SpA groups (*P* = .942) (Table 1).

Among RA patients, 87 were receiving b/tsDMARDs, whereas 120 patients with SpA were under similar therapy. There was no statistically significant difference in the use of b/tsDMARDs between the 2 groups (*P* = .243). A proportion of patients was treated with either a biologic or a targeted synthetic DMARD concomitantly with a csDMARD. The medical treatments of the patients are shown in Table 2.

When all participants were stratified according to the use of b/tsDMARDs, 207 patients (57.5%) were receiving b/tsDMARDs, whereas 153 patients (42.5%) were not. Among those treated with b/tsDMARDs, 42 patients (20.3%) were FM positive and 165 (79.7%) were FM negative. In contrast, among the untreated group, 46 patients (30.1%) were FM positive and 107 (69.9%) were FM negative. The prevalence of FM was significantly higher in patients not receiving b/tsDMARDs compared to those under this treatment (*P* = .033).

On the other hand, when structural impairment was evaluated using the BASMI, patients receiving b/tsDMARDs demonstrated higher mean scores (1.39 ± 2.35) compared to those not receiving such therapy (0.52 ± 1.28), and this difference was statistically significant (*P* = .001).

Within the SpA subgroup, 98 patients were not treated with b/tsDMARDs, while 120 received one of these agents. FM was present in 27 patients in the untreated group and 26 patients in the treated group. The difference in FM prevalence between these subgroups was not statistically significant (*P* = .314).

When patients with FM in the SpA group were compared according to the use of b/tsDMARDs, it was determined that 26 of the 53 patients were receiving b/tsDMARDs, while 27 were not (Table 2). When these patients were compared in terms of FIQ and BASDAI scores, the mean FIQ score was 59.77 ± 15.24 in patients not receiving b/tsDMARDs, whereas it was 53.44 ± 15.83 in those receiving b/tsDMARDs. There was no statistically significant difference between the 2 groups in terms of FIQ scores (*P* = .152). However, when the groups were compared regarding BASDAI scores, the mean BASDAI score was 5.60 ± 1.75 in FM patients not using b/tsDMARDs and 4.48 ± 1.89 in those using them. A statistically significant difference was observed between the groups in terms of BASDAI scores (*P* = .029).

In the RA subgroup, of 142 patients, 55 were not treated with b/tsDMARDs, whereas 87 received these agents. FM was identified in 19 untreated and 16 treated patients. The prevalence of FM was higher among RA patients not receiving b/tsDMARDs compared to those under treatment (*P* = .030) (Table 2).

Among the 35 patients with RA who had concomitant FM, 16 were receiving b/tsDMARDs therapy, while 19 were not. The median FIQ score was calculated as 60 (interquartile range [IQR]: 47.50-67.25) in FM patients not receiving b/tsDMARDs therapy, and 53.50 (IQR: 35.25-76.00) in those receiving it. There was no statistically significant difference between the 2 groups in terms of FIQ scores (*P* = .717).

On the other hand, when FM-positive and -negative patients in the RA group were compared regarding DAS28 scores, the median DAS28 value was 3.00 (IQR: 2.60-4.30) in patients not receiving b/tsDMARDs, and 2.75 (IQR: 2.30-3.90) in those on b/tsDMARDs therapy. No statistically significant difference was observed between the 2 groups in terms of median DAS28 scores (*P* = .266).

**Figure 1.**
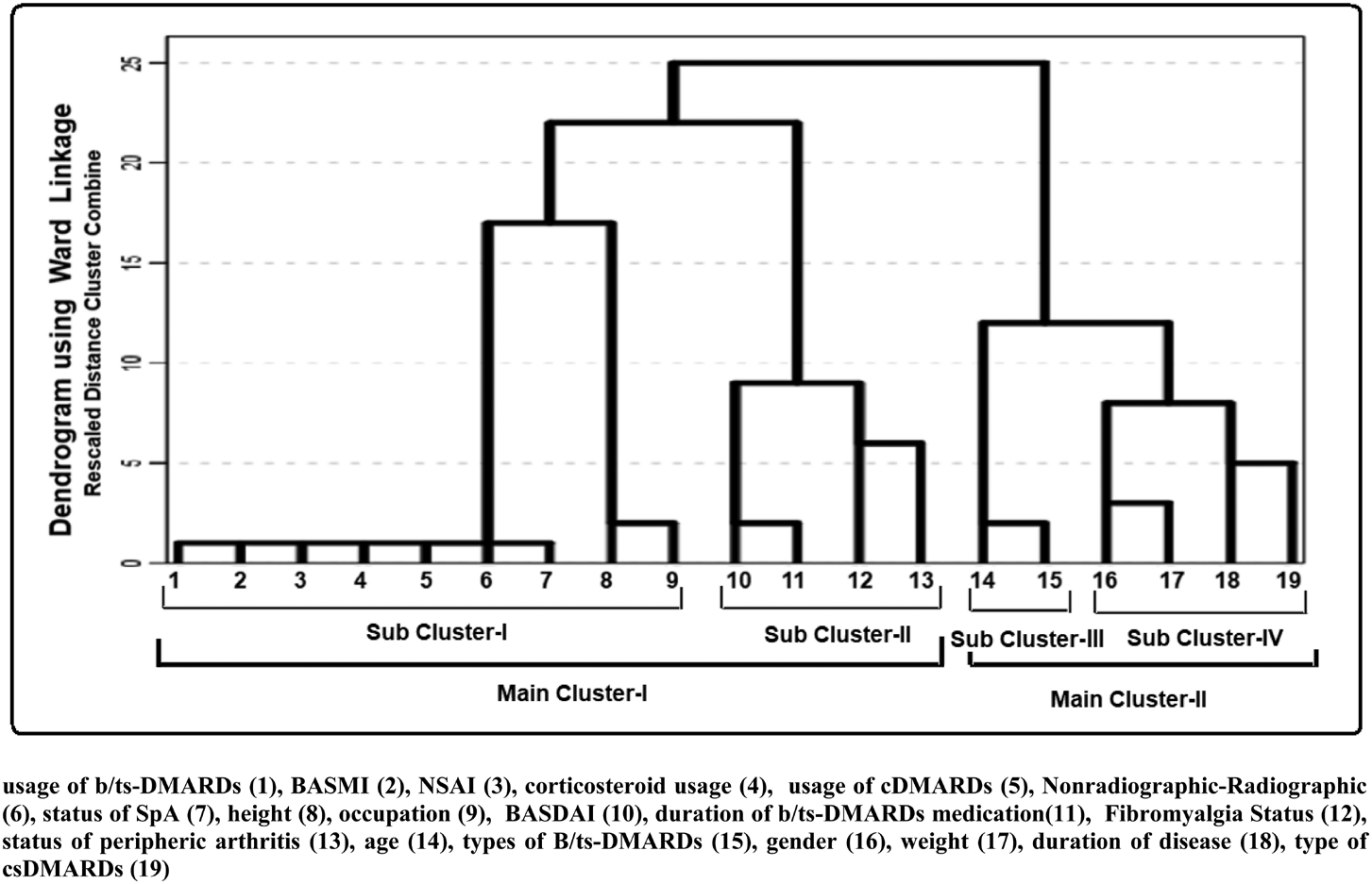
Dendrogram showing variable clusters and the relationships between clinical parameters found with the “Hierarchical Clustering Method” for the spondyloarthritis group. Usage of b/tsDMARDs (1), BASMI (2), NSAID (3), corticosteroid usage (4), usage of cDMARDs (5), Nonradiographic-Radiographic (6), status of SpA (7), height (8), occupation (9), BASDAI (10), duration of b/tsDMARDs medication (11), Fibromyalgia Status (12), status of peripheral arthritis (13), age (14), types of b/tsDMARDs (15), gender (16), weight (17), duration of disease (18), type of csDMARDs (19).

**Figure 2.**
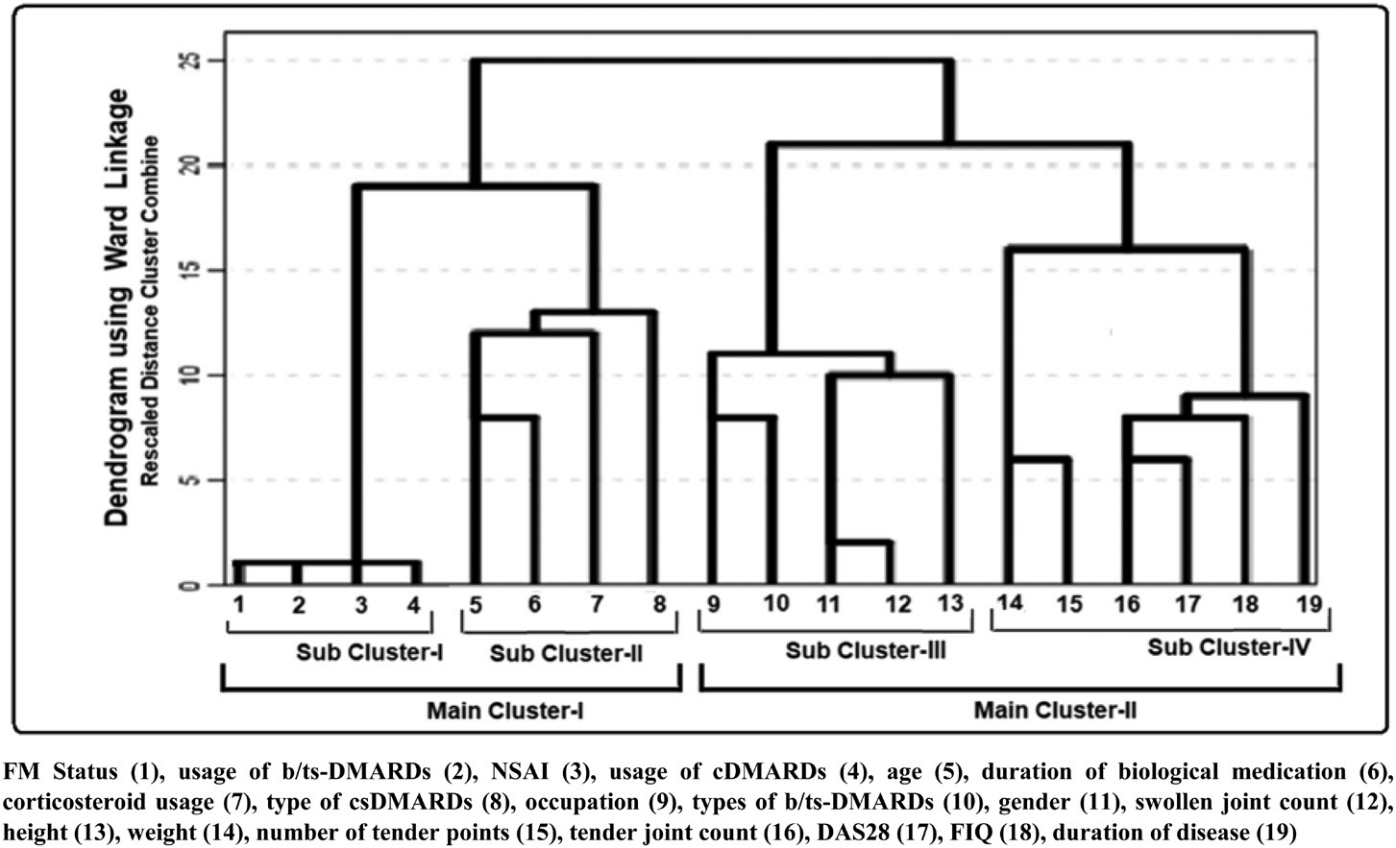
A dendrogram showing clusters and relationships of parameters generated using the “Hierarchical Clustering Method” for patients with RA. Fibromyalgia status (1), usage of b/tsDMARDs (2), NSAID (3), usage of cDMARDs (4), age (5), duration of biological medication (6), corticosteroid usage (7), type of csDMARDs (8), occupation (9), types of b/tsDMARDs (10), gender (11), swollen joint count (12), height (13), weight (14), number of tender points (15), tender joint count (16), DAS28 (17), FIQ (18), duration of disease (19).

A dendrogram illustrating the clusters and interrelationships among the studied parameters, generated using the hierarchical clustering method, is presented in Figures 1 and 2.

Hierarchical cluster analysis is a multivariate technique that simultaneously considers all variables to explore patterns of similarity or association. The resulting dendrogram offers a visual representation of how variables or observations group together, helping to interpret their relative proximity.

According to the dendrogram for patients with spondyloarthritis, subcluster II demonstrates a close relationship among BASDAI scores, duration of b/tsDMARD therapy, FM status, and the presence of peripheral arthritis. This pattern indicates a strong association between the duration of b/tsDMARDs use and FM status (Figure 1).

According to the dendrogram for patients with RA, subcluster I indicates a close relationship between FM status, usage of b/tsDMARDs, usage of non-steroidal anti-inflammatory drugs, and usage of csDMARDs. This analysis indicates a strong association between FM status and the usage of b/tsDMARDs (Figure 2).

## Discussion

In this study, the prevalence of FM did not differ significantly between patients with SpA and those with RA. When participants were stratified according to b/tsDMARDs agent use, however, FM was more common among patients who were not receiving b/tsDMARDs therapies. A similar pattern emerged within the RA subgroup: individuals not treated with b/tsDMARDs demonstrated a higher prevalence of FM, suggesting a meaningful relationship between the use of biologic therapies and FM status in RA. Prior research has consistently described the co-occurrence of FM across a range of rheumatic disorders, with reported prevalence rates exceeding those observed in the general population.^29^^,^^30^ One explanatory hypothesis is that persistent nociceptive input generated by rheumatic inflammation promotes central nervous system hyperexcitability and lowers pain thresholds, leading to FM-like symptomatology.^31^ This hypothesis aligns with the concept that heightened inflammatory activity may precipitate or exacerbate central sensitization,^32^ while dampening inflammatory pathways may attenuate chronic centrally mediated pain and thereby reduce FM severity.^33^ Evidence for the involvement of neuroinflammatory mechanisms is supported by elevated serum concentrations of cytokines such as IL-8, IL-6, TNF-α, IL-17, and IL-1β, as well as by the relatively high occurrence of FM in inflammatory arthritis, which together indicate that peripheral inflammation may amplify nociceptive signaling and contribute to central sensitization processes.^34^^,^^35^

Although FM in the context of inflammatory rheumatic diseases has been increasingly explored, 2 key questions remain insufficiently resolved: (i) what is the true prevalence of FM in patients with inflammatory rheumatic conditions, and (ii) how does biologic therapy influence FM when the 2 disorders coexist?^34^ Bello et al^36^ documented an FM prevalence of 21.3% among axSpA patients meeting ASAS imaging criteria, a value similar to the present SpA cohort. Abbasi et al^37^ likewise reported an FM prevalence of 25.83% among 120 RA patients. In parallel, a meta-analysis by Duffield and colleagues^38^ estimated a pooled FM prevalence of 21% in RA, consistent with the present results.

Macfarlane et al,^39^ analyzing data from a national axSpA registry, observed symptomatic improvement in patients with concomitant FM following TNF-α inhibitor therapy. Molto et al^40^ similarly demonstrated a decline in FM prevalence over time in patients treated with anti-TNF agents. In the current SpA group, a strong association was identified between the duration of b/tsDMARD exposure and FM status (Figure 1). Fibromyalgia-positive individuals treated with b/tsDMARDs displayed notably lower BASDAI scores compared with those not receiving such therapies. Among RA patients, a similar association emerged between FM status and b/tsDMARDs usage (Figure 2). Although biologic-treated RA patients with FM exhibited lower FIQ and DAS28 values than non-users, statistical significance was not reached, implying that biologic therapies may still exert a moderating effect on FM severity.

The frequency of FM was lower among RA patients undergoing biologic treatment. Comparable findings were reported by Rotondo et al,^33^ who followed 64 RA and Psoriatic arthritis (PsA) patients treated with b/tsDMARDs for 6 months: FM was no longer identifiable in 16 of 50 FM-positive individuals at baseline (32%), and patients experienced marked improvements in FM-related indices, including the widespread pain index and symptom severity scale. These findings are substantially consistent with the current results. In both SpA and RA subgroups, FM-positive individuals using b/tsDMARDs had lower FIQ scores than non-users, although without statistical significance.

The absence of a difference in FM prevalence between biologic-treated and untreated SpA patients may be influenced by the sex distribution, as FM is more common among women, and RA disproportionately affects women compared with SpA.^41^^,^^42^

In RA, higher DAS28 and The Health Assessment Questionnaire (HAQ) scores have been previously documented among patients with coexisting FM (43). In line with these reports, Chakr et al^43^ found increased DAS28 values in RA patients with FM relative to those without. The present findings parallel this pattern, as FM-positive RA patients exhibited significantly higher DAS28 scores.

FM is characterized primarily by widespread musculoskeletal pain, frequently accompanied by fatigue, sleep disturbances, mood alterations, stiffness, and cognitive difficulties.^44^ Several of these symptoms overlap with clinical features of inflammatory rheumatic diseases. Neurochemical alterations, including changes in serotonin pathways, have been proposed as potential mediators linking inflammatory diseases and FM.^45^ Such overlap may artificially inflate disease activity assessments in FM-positive individuals with inflammatory arthritis, which could influence clinical decisions regarding biologic therapy.^46^ Notably, biologic DMARDs may ameliorate FM-related symptoms in some patients with inflammatory arthritis.^34^

Neuroinflammation is increasingly recognized as a central mechanism in FM pathophysiology. Elevated concentrations of pro-inflammatory cytokines in serum and cerebrospinal fluid, along with the high prevalence of FM in chronic inflammatory arthritis, suggest that peripheral inflammation can potentiate nociceptive transmission and contribute to chronic pain via central sensitization.^34^^,^^35^ Additionally, increases in pro-inflammatory cytokines are sometimes accompanied by reductions in anti-inflammatory mediators such as IL-4 and IL-13,^14^^,47^ leading to broader immune dysregulation. This includes elevations in neutrophil-to-lymphocyte ratios and abnormalities in T-cell subsets—particularly CD4^+^ and natural killer T-cell populations.^48^^,^^49^ Thus, the interplay between FM and inflammation likely extends beyond altered pain processing, potentially involving dysregulated immune pathways that sustain a cycle of chronic pain, inflammation, and immune imbalance.^1^^,^^9^^,^^33^^,^^50^ These mechanisms align closely with the patterns observed in the present cohort.

In summary, FM was less frequent among RA patients treated with b/tsDMARDs, and those receiving this therapy may result in lower FIQ scores. Fibromyalgia-positive individuals had higher BASDAI and DAS28 scores, yet these measures tended to be lower in patients concurrently receiving b/tsDMARD treatment. Further controlled studies with larger patient populations are needed to evaluate the short- and long-term effects of b/tsDMARD therapy on FM in patients with RA and SpA.

## Figures and Tables

**Table 1.** Demographic and Clinical Characteristics of Patients in Spondyloarthritis and Rheumatoid Arthritis Groups

	**SpA ** **(n = 218)**	**RA ** **(n = 142)**	*P*
Age (years)	39.02 ± 11.52	50.80 ± 12.78	**.001**
Gender (F/M)	116/102	110/32	**.001**
Height	167.86 ± 7.49	163.81 ± 7.94	**.001**
Weight	72.55 ± 11.88	70.37 ± 13.43	.107
Duration of disease (years)	9.27 ± 7.71	8.24 ± 7.33	.203
Duration of biologics and targeted synthetic drug traetment (years)	3.24 ± 2.90	2.86 ± 2.47	.304
Tender point count	12.87 ± 1.55	12.20 ± 1.99	.082
FIQ	56.67 ± 15.70	56.62 ± 17.11	.662
DAS28	–	2.50 ± 1.32	
BASDAI	3.27 ± 2.04	–	
BASMI	1.00 ± 1.99	–	
Presence of FM, n (%)	53/218 (24.3)	35/142 (24.6)	.942

BASDAI, Bath Ankylosing Spondylitis Disease Activity Index; BASMI, Bath Ankylosing Spondylitis Metrology Index; DAS, Disease Activity Score; F, female; FIQ, Fibromyalgia Impact Questionnaire; FM, fibromyalgia; M, male; RA, rheumatoid arthritis; SpA, spondyloarthritis.

**Table 2.** Medication and Disease Activity Scores in Both Groups with Fibromyalgia and Without Fibromyalgia

	**SpA ** **(n = 218)**		*P*	**RA ** **(n = 142)**		*P*
	**FM Positive** **(n = 53)**	**FM Negative** **(n = 165)**		**FM Positive** **(n = 35)**	**FM Negative** **(n = 107)**	
Female/Male	48/5	68/97		34/1	76/31	
Medication						
csDMARDs, n	5	17	.546	29	90	.861
Corticosteroids, n	–	–		18	75	**.044**
NSAIDs, n	31	83	.299			
Biologics or tsDMARDs, n	26	94	.314	16	71	**.030**
Anti-TNF, n	20	74	.146	8	46	.245
Sekukinumab, n	4	16		–	–	
Ixekizumab, n	2	–		–	–	
Upadacitinib, n	–	3		2	3	
Tofacitinib, n	–	1		0	3	
Rituximab, n	–	–		0	3	
Abatacept, n	–	–		5	7	
Baricitinib, n	–	–		0	2	
Tocilizumab, n	–	–		1	8	
Duration of disease	8.70 ± 6.49	9.45 ± 8.07	.543	10.34 ± 9.30	7.55 ± 6.49	.050
Duration of biologics and targeted synthetic drug treatment	2.93 ± 2.15	3.32 ± 3.07	.548	3.35 ± 2.21	2.75 ± 2.16	.320
BASDAI (mean ± SD)	5.05 ± 1.89	2.70 ± 1.74	**.001**	–	–	
BASMI (mean ± SD)	0.75 ± 1.29	1.08 ± 2.16	.189	–	–	
DAS28 (mean ± SD)	–	–		3.01 ± 1.16	2.34 ± 1.33	**.009**
FIQ (mean ± SD)	56.67 ± 15.70			56.62 ± 17.11		

BASDAI, Bath Ankylosing Spondylitis Disease Activity Index; BASMI, Bath Ankylosing Spondylitis Metrology Index; csDMARDs, conventional synthetic disease-modifying antirheumatic drugs; DAS, Disease Activity Score; FIQ, Fibromyalgia Impact Questionnaire; FM, fibromyalgia; NSAIDs, non-steroidal anti-inflammatory drugs; RA, rheumatoid arthritis; SpA, spondyloarthritis; TNF, tumor necrosis factor; tsDMARDs, targeted synthetic disease-modifying antirheumatic drugs.

## Data Availability

The data that support the findings of this study are available on request from the corresponding author.
